# Mitofusin-2 acts as biomarker for predicting poor prognosis in hepatitis B virus related hepatocellular carcinoma

**DOI:** 10.1186/s13027-018-0212-7

**Published:** 2018-11-26

**Authors:** Xiumei Wang, Youde Liu, Jing Sun, Wenjing Gong, Ping Sun, Xiangshuo Kong, Miaomiao Yang, Weiwei Zhang

**Affiliations:** 1 Department of Oncology, The Affiliated Yantai Yuhuangding Hospital of Qingdao University, Yantai, Shandong 264000 People’s Republic of China; 2 Department of Hepatology, Infectious Disease Hospital of Yantai City, Yantai, Shandong 264001 People’s Republic of China

**Keywords:** Mitofusin-2, Prognosis, Hepatocellular carcinoma, Biomarker

## Abstract

**Objective:**

To investigate the expression of Mitofusin-2 (MFN2) in HCC tissues and its role in the development of HCC.

**Methods:**

A total of 107 HCC specimens were collected for tissue microarray analysis and immunohistochemistry (IHC) analysis. The relationship between MFN2 expression and clinical features of patients with HCC was analyzed.

**Results:**

Expression level of MFN2 in HCC tissues was 0.92 ± 0.78, significantly lower than that of matched paracancerous liver tissues (1.25 ± 0.75). Patients with low expression of MFN2 had significantly higher rates of cirrhosis than those with high expression of MFN2 (*P* = 0.049). Kaplan-Meier survival analysis showed that HCC patients with low expression of MFN2 had a worse prognosis in overall survival than HCC patients with high expression of MFN2 (*P* = 0.027). Patients with high expression of MFN2 had a better prognosis in disease-free survival compared with HCC patients with low expression of MFN2 (*P* = 0.047). Vascular invasion and MFN2 expression were shown to be prognostic variables for overall survival in patients with HCC. Multivariate analysis showed that vascular invasion (*P* < 0.001) and MFN2 expression (*P* = 0.045) were independent prognostic factors for overall survival. Vascular invasion (*P* < 0.001) and MFN2 expression (*P* = 0.042) were independent risk factors associated with disease-free survival.

**Conclusion:**

Our data revealed that MFN2 expression was decreased in HCC samples. High MFN2 expression was correlated with longer survival times in patients with HCC and served as an independent factor for better outcomes. Our study therefore provides a promising biomarker for the prognostic prediction of HCC and a potential therapeutic target for the disease.

## Introduction

Hepatocellular carcinoma (HCC) is one of the most common malignancies in the world [1–3]. Approximately 437,000 people are diagnosed with HCC each year worldwide, of which approximately 50% occur in China [4, 5]. Although the therapeutic methods of HCC have improved with advances in surgical methods, interventions, ablation, etc., the 5-year survival rate of patients is very low [6, 7]. In China, HBV infection is the most important etiology of HCC [8–11]. Nearly half of HCC patients are infected with HBV, and the geographical distribution of HBV infection is also highly correlated to the geographical distribution of HCC [12–14]. However, the molecular mechanisms by which HBV infection induces HCC remain unclear. Therefore, understanding the mechanism of hepatitis B-related HCC development is particularly important for early screening, clinical diagnosis and prevention of HCC.

Mitochondrial fusion protein 2 (MFN2) is localized on the mitochondrial outer membrane and is involved in the regulation of fusion of mitochondrial outer membrane [15, 16]. MFN2 promotes cellular apoptosis and inhibits cell proliferation [17, 18]. Studies showed that the expression of MFN2 in colorectal cancer tissues and breast cancer tissues is significantly lower than that in normal adjacent tissues [19, 20]. These results suggest that MFN2 plays an important role in the occurrence and development of malignant tumors. However, the role of MFN2 in liver cancer has not been reported yet.

This study was designed to explore the expression level of MFN2 in the cancer tissues of patients with hepatitis B-related HCC, analyze the relationship between the expression level of MFN2 and clinicopathological features, and investigate its role in the regulation of hepatitis B-related HCC.

## Subjects and methods

### Subjects

We collected a total of 107 HCC tissues from August 2000 to Jun 2010. All patients were sero-positive of hepatitis B surface antigen. None of the patients received any chemotherapy or radiotherapy before collection of the tissue. The follow-up period was defined as the time interval between the date of operation and the date of death or the last follow-up. The study was approved by the medical ethics committee of Yantai Yuhuangding Hospital. Since all specimens used were anonymous, the Medical Ethics Committee exempted patients from the need for informed consent.

### Tissue microarray construction and immunohistochemistry

Tissue microarray (TMA) was constructed. Each tissue core (diameter: 0.6 mm) was perforated and re-embedded from the labeled area by using a tissue array. The specimens were fixed with 4% paraformaldehyde. The biotin blocking Kit (Dark, Germany) was closed. After closure, the tissues were incubated with MFN2 antibodies (#9482, 1:1000, CST, USA) in a humid chamber at 4 °C for the night. The tissues were washed with PBS three times and incubated with biotinylated goat anti-rabbit antibodies for 1 hour. Finally, the slices were stained with hematoxylin and observed under a microscope. Semi-quantitative IHC was used to assess MFN2 protein expression levels according to the following criteria: “0” (negative staining), “1” (weak staining), “2” (moderate staining) and “3” (strong staining). The final score was calculated as the percentage of positive expression multiplied by the intensity score. The median IHC score was used as a cut-off value for determining high and low levels of expression.

### Statistical analysis

Statistical analysis was performed using SPSS software (version 13; SPSS Inc., Chicago, IL, USA). Student’s t test or Chi square test was used to examine the correlation between MFN2 expression and clinical and pathological variables. The Kaplan-Meier method (logarithmic rank test) was used to construct the survival curve. Multivariate Cox proportional hazards regression model was used to assess the independent predictive value of MFN2. *P* value less than 0.05 was defined as statistically significant.

## Results

### Expression of MFN2 in the HCC TMA

**Fig. 1:**
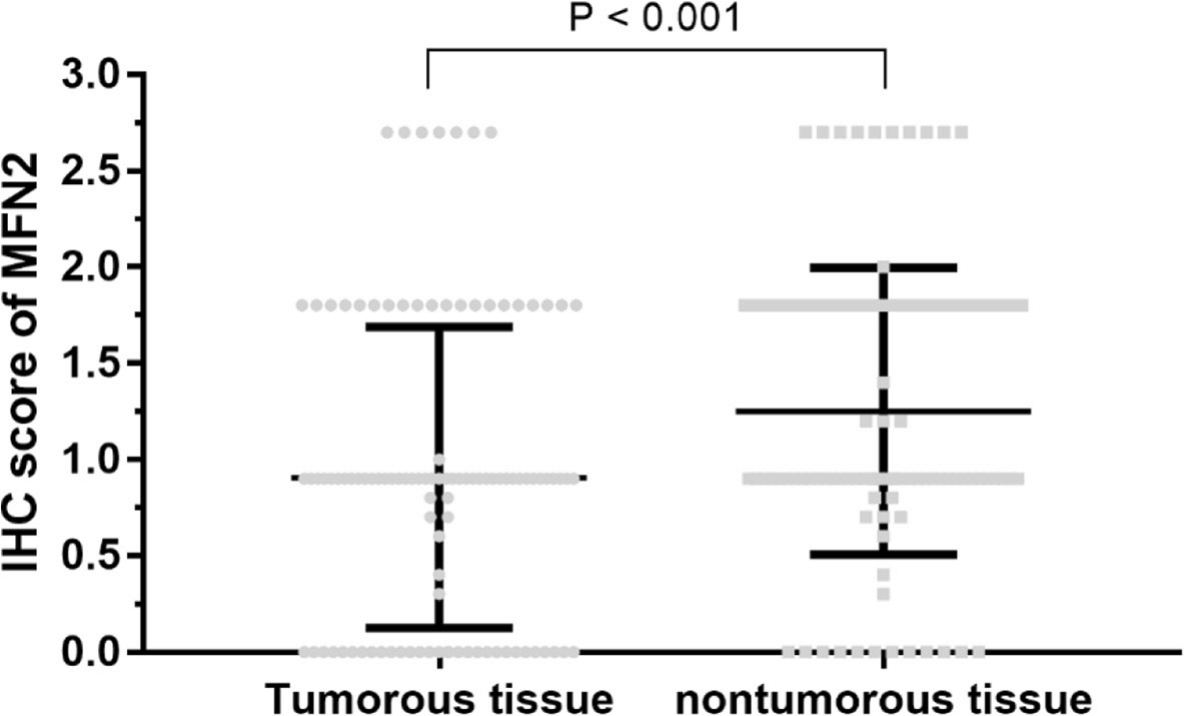
Expression of MFN2 in HCC tissues. The MFN2 IHC score for HCC tissue was 0.92 ± 0.78, significantly higher than that of matched nontumorous tissue (1.25 ± 0.75, *P* < 0.001)

The expression level of MFN2 in 107 pairs of HCC tissues and matched paracancerous liver tissues were measured by IHC staining. The results showed that the expression level of MFN2 in HCC tissues was 0.92 ± 0.78, significantly lower than that of matched paracancerous liver tissues (1.25 ± 0.75, *P* < 0.001, Fig. 1).

### Association of cytoplasmic MFN2 with HCC clinical features

According to the median score (0.9) of MFN2 expression in HCC tissues, all enrolled HCCs were divided into MFN2 low expression group (MFN < 0.9) and MFN2 high expression group (MFN ≥ 0.9). Demographic data and clinical features of the patients in the two groups were compared. We found that patients with low expression of MFN2 had significantly higher rates of cirrhosis than those with high expression of MFN2 (*P* = 0.049), as shown in Table 1.

**Table 1:** Clinical variables in patients with high and low expression of MFN2

Variable	MFN2 expression		*P* value
	High expression	Low expression	
Sample size	33	74	
Age, years			0.179
> 50	15 (45.5%)	44 (59.5%)	
≤ 50	18 (54.5%)	30 (40.5%)	
Gender			0.844
Male	29 (87.9%)	64 (86.5%)	
Female	4 (12.1%)	10 (13.5%)	
AFP, ng/mL			0.420
< 20	10 (30.3%)	17 (23.0%)	
≥ 20	23 (69.7%)	57 (77.0%)	
Cirrhosis			0.049
Yes	30 (90.9%)	55 (74.3%)	
No	3 (9.1%)	19 (25.7%)	
Tumor size, cm			0.128
< 5	9 (27.3%)	11 (14.9%)	
≥ 5	24 (72.7%)	63 (85.1%)	
Differentiation			0.202
Well-moderate	6 (18.2%)	7 (9.5%)	
Poor-undifferentiated	27 (81.8%)	67 (90.5%)	
TNM stage			0.196
I–II	15 (45.5%)	24 (32.4%)	
III–IV	18 (54.5%)	50 (67.6%)	
Vascular invasion			0.192
Yes	4 (12.1%)	17 (23.0%)	
No	29 (87.9%)	57 (77.0%)	

*Abbreviations***:** *MFN2* Mitofusin-2, *AFP* alpha-fetoprotein

There are 107 paratumor tissues we have collected and enrolled, 85 of them were diagnosed with cirrhosis and 22 were not diagnosed with cirrhosis. The MFN2 IHC scores were 1.27 ± 0.75 in cirrotic tissue and 1.18 ± 0.74 in non-cirrotic tissue (*P* = 0.605).

### Association of MFN2 expression with clinical outcomes in patients with HCC

**Fig. 2:**
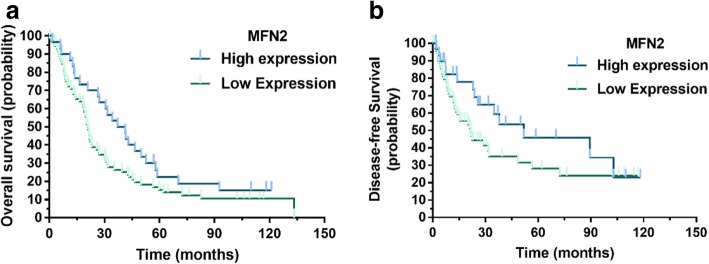
The prognostic predictive value of MFN2 in HCC. Kaplan-Meier analysis revealed that patients with low expression of MFN2 had significantly worse outcomes in terms of overall survival (*P* = 0.027, **a**). Compared with the patients with high MFN2 expression, patients with low MFN2 expression had a significantly worse disease-free survival (*P* = 0.047, **b**)

To determine the prognostic value of MFN2 in HCC, we performed a Kaplan-Meier survival analysis. The results showed that HCC patients with low expression of MFN2 had a worse prognosis in overall survival than patients with high expression of MFN2 (*P* = 0.027). We also compared the difference in the disease-free survival between the MFN2 high-expression group and the low-expression group. The results showed that patients with high expression of MFN2 had a better prognosis in disease-free survival compared with patients with low expression of MFN2 (*P* = 0.047), as shown in Fig. 2.

### Univariate and multivariate analyses of prognostic factors in HCC

To evaluate whether MFN2 expression was an independent risk factor for outcomes in HCC, both univariate and multivariate analyses were conducted. The vascular invasion and MFN2 expression were shown to be prognostic factors for overall survival in patients with HCC. The multivariate analysis showed that vascular invasion (*P* < 0.001) and MFN2 expression (*P* = 0.045) were independent prognostic factors for overall survival (Table 2).

**Table 2:** Univariate and multivariate analyses of variables for overall survival

Variables	Univariate analysis			Multivariate analysis		
	HR	95% CI	*P*	HR	95% CI	*P*
Age, years	1.132	0.746–1.719	0.559			
Sex	0.739	0.382–1.429	0.368			
AFP	1.223	0.760–1.968	0.406			
Cirrhosis	0.835	0.507–1.375	0.478			
Tumor size, cm	1.237	0.720–2.128	0.441			
Differentiation	1.054	0.573–1.939	0.867			
TNM stage	0.978	0.634–1.510	0.921			
Vascular invasion	2.884	1.722–4.832	< 0.001	2.169	1.167–3.628	< 0.001
MFN2 expression	1.528	1.060–2.433	0.032	1.444	1.012–1.799	0.045

*Abbreviations***:** *MFN2* Mitofusin-2, *AFP* alpha-fetoprotein

We further explored the risk factors associated with disease-free survival (Table 3). Univariate analysis showed that vascular invasion and MFN2 expression were risk factors associated with disease-free survival. Multivariate analysis also showed that vascular invasion (*P* < 0.001) and MFN2 expression (*P* = 0.042) were independent risk factors associated with disease-free survival.

**Table 3:** Univariate and multivariate analyses for disease-free survival

Variables	Univariate analysis			Multivariate analysis		
	HR	95% CI	*P*	HR	95% CI	*P*
Age, years	0.999	0.982–1.017	0.920			
Sex	0.739	0.382–1.429	0.368			
AFP	1.223	0.760–1.968	0.406			
Cirrhosis	0.835	0.507–1.375	0.478			
Tumor size, cm	1.237	0.720–2.128	0.441			
Differentiation	1.056	0.573–1.945	0.861			
TNM stage	0.976	0.632–1.508	0.912			
Vascular invasion	2.884	1.722–4.832	< 0.001	2.703	1.603–4.599	< 0.001
MFN2 expression	1.528	1.062–2.433	0.034	1.360	1.048–2.181	0.042

*Abbreviations*: *MFN2* Mitofusin-2, *AFP* alpha-fetoprotein

## Discussion

Mitochondria are involved in the regulation of cellular energy metabolism, signaling, and programmed cell death [21, 22]. The shape and size of mitochondria are regulated by complex and sophisticated systems [23, 24]. MFN2 is a protein located in the outer membrane of mitochondria and is mainly involved in the regulation of fusion of mitochondrial outer membrane [15, 17]. HCC is a disease with significant heterology due to the various etiology and other mechanism [25, 26]. There is growing evidence showing that MFN2 is involved in a range of pathologies, including tumorigenesis, diabetes, cardiovascular disease and so on [15, 17, 18]. In this study, we found that MFN2 is downregulated in HCC and the low expression is associated with poor prognosis. The results provided direction for future research of HCC mitochondrial dysfunction, and MFN2 may serve as a promising biomarker for HCC.

MFN2 is located at the 36.22 locus of human chromosome 1 short arm. Cytogenetic analysis found that human chromosome 1 short arm 3 region 6 is the mutation-prone area of many malignant tumors [15, 17, 18]. Therefore, MFN2 is suspected to be a tumor suppressor gene. The results of this study showed that MFN2 is expressed at low level in liver cancer and is associated with poor prognosis, suggesting that MFN2 is a tumor suppressor gene in HCC. We also found that MFN2 is associated with cirrhosis. Whether MFN2 is causative to cirrhosis requires further research.

Previous studies have shown that MFN2 plays an important anti-proliferative effect in breast cancer cells [27, 28]. MFN2 significantly inhibit the proliferation of breast cancer cells in vitro and increase the chemo-sensitivity of cancer cells to induce their apoptosis [27, 28]. In addition, studies also showed that exogenous MFN2 can inhibit the proliferation of gastric cancer cells and induce apoptosis in vitro by promoting the flow of calcium ions from the endoplasmic reticulum to mitochondria, thereby disrupting the mitochondrial calcium ion homeostasis [29, 30]. However, the role of MFN2 in HCC is still unclear. This study demonstrated that low expression of MFN2 is closely related to the poor prognosis of patients with HCC, and the specific molecular mechanism warrants further study. In addition, since all patients enrolled in our study were HBV-related HCC patients. Therefore, we conclude that MFN2 can be used as a prognostic biomarker for HBV-related HCC. However, for HCV or non-viral related HCC, whether MFN2 can be used as a biomarker for prognosis still needs further study. Since in China, the majority of HCC is caused by chronic HBV infection. Therefore, exploring the expression level and significance of MFN2 in HCC caused by other etiologies still needs a multi-center clinical study to confirm.

There are some limitations in this study. Firstly, the sample size is relatively small, so the results may be biased. Secondly, the data collected in this study came from a single center and this may lead to some enrollment bias. A multicenter prospective study is needed to further validate the role of MFN2 in HCC and its potential prognosis prediction value.

In summary, our study results revealed a role of MFN2 in the development of HCC. Our data showed that MFN2 expression was decreased in HCC samples. High MFN2 expression was also correlated with longer survival times in patients with HCC and served as an independent predictor for better outcomes. Collectively, our data suggest that MFN2 is a promising biomarker for the prognosis of patients with HCC and a potential target in HCC treatment.

## References

[1] Forner A, Llovet JM, Bruix J (2012). Hepatocellular carcinoma. LANCET.

[2] Forner A, Reig M, Bruix J. Hepatocellular carcinoma. LANCET. 2018. 10.1016/S0140-6736(18)30010-2 29307467

[3] Cai SH, Lu SX, Liu LL, Zhang CZ, Yun JP (2017). Increased expression of hepatocyte nuclear factor 4 alpha transcribed by promoter 2 indicates a poor prognosis in hepatocellular carcinoma. Therap Adv Gastroenterol.

[4] Clavien PA, Lesurtel M, Bossuyt PM, Gores GJ, Langer B, Perrier A (2012). Recommendations for liver transplantation for hepatocellular carcinoma: an international consensus conference report. LANCET ONCOL.

[5] El-Serag HB, Kanwal F (2014). Epidemiology of hepatocellular carcinoma in the United States: where are we? Where do we go?. HEPATOLOGY.

[6] Ho SY, Liu PH, Hsu CY, Chiou YY, Su CW, Lee YH, Huang YH, Lee FY, Hou MC, Huo TI (2018). Prognostic performance of ten liver function models in patients with hepatocellular carcinoma undergoing radiofrequency ablation. Sci Rep.

[7] Ikeda K, Saitoh S, Tsubota A, Arase Y, Chayama K, Kumada H, Watanabe G, Tsurumaru M (1993). Risk factors for tumor recurrence and prognosis after curative resection of hepatocellular carcinoma. CANCER-AM CANCER SOC.

[8] Cai SH, Lv FF, Zhang YH, Jiang YG, Peng J (2014). Dynamic comparison between Daan real-time PCR and Cobas TaqMan for quantification of HBV DNA levels in patients with CHB. BMC Infect Dis.

[9] Cai S, Cao J, Yu T, Xia M, Peng J (2017). Effectiveness of entecavir or telbivudine therapy in patients with chronic hepatitis B virus infection pre-treated with interferon compared with de novo therapy with entecavir and telbivudine. Medicine (Baltimore).

[10] Xiao YB, Cai SH, Liu LL, Yang X, Yun JP (2018). Decreased expression of peroxisome proliferator-activated receptor alpha indicates unfavorable outcomes in hepatocellular carcinoma. Cancer Manag Res.

[11] Zeng J, Cai S, Liu J, Xue X, Wu X, Zheng C (2017). Dynamic changes in liver stiffness measured by transient Elastography predict clinical outcomes among patients with chronic hepatitis B. J Ultrasound Med.

[12] Cai S, Li Z, Yu T, Xia M, Peng J (2018). Serum hepatitis B core antibody levels predict HBeAg seroconversion in chronic hepatitis B patients with high viral load treated with nucleos(t)ide analogs. INFECT DRUG RESIST.

[13] Ou H, Cai S, Liu Y, Xia M, Peng J (2017). A noninvasive diagnostic model to assess nonalcoholic hepatic steatosis in patients with chronic hepatitis B. Therap Adv Gastroenterol.

[14] Wu X, Cai S, Li Z, Zheng C, Xue X, Zeng J, Peng J (2016). Potential effects of telbivudine and entecavir on renal function: a systematic review and meta-analysis. Virol J.

[15] Stuppia G, Rizzo F, Riboldi G, Del BR, Nizzardo M, Simone C, Comi GP, Bresolin N, Corti S (2015). MFN2-related neuropathies: clinical features, molecular pathogenesis and therapeutic perspectives. J Neurol Sci.

[16] Yu HY, Guo YH, Gao W (2010). Mitochondrial fusion protein Mfn2 and cardiovascular diseases. Sheng Li Ke Xue Jin Zhan.

[17] Xu K, Chen G, Li X, Wu X, Chang Z, Xu J, Zhu Y, Yin P, Liang X, Dong L (2017). MFN2 suppresses cancer progression through inhibition of mTORC2/Akt signaling. Sci Rep.

[18] Xue R, Meng Q, Lu D, Liu X, Wang Y, Hao J (2018). Mitofusin2 induces cell autophagy of pancreatic Cancer through inhibiting the PI3K/Akt/mTOR signaling pathway. Oxidative Med Cell Longev.

[19] Cheng X, Zhou D, Wei J, Lin J (2013). Cell-cycle arrest at G2/M and proliferation inhibition by adenovirus-expressed mitofusin-2 gene in human colorectal cancer cell lines. NEOPLASMA.

[20] Li Y, Dong W, Shan X, Hong H, Liu Y, Liu Y, Liu X, Zhang X, Zhang J (2018). The anti-tumor effects of Mfn2 in breast cancer are dependent on promoter DNA methylation, the P21(Ras) motif and PKA phosphorylation site. Oncol Lett.

[21] Bonora M, Wieckowski MR, Sinclair DA, Kroemer G, Pinton P, Galluzzi L. Targeting mitochondria for cardiovascular disorders: therapeutic potential and obstacles. Nat Rev Cardiol. 2018. 10.1038/s41569-018-0074-0 PMC6349394 30177752

[22] Chakrabarty S, Kabekkodu SP, Singh RP, Thangaraj K, Singh KK, Satyamoorthy K. Mitochondria in health and disease. MITOCHONDRION. 2018. 10.1016/j.mito.2018.06.006 29944924

[23] Anderson RG, Ghiraldeli LP, Pardee TS. Mitochondria in cancer metabolism, an organelle whose time has come? Biochim Biophys Acta. 2018. 10.1016/j.bbcan.2018.05.005 PMC6420819 29807044

[24] Angajala A, Lim S, Phillips JB, Kim JH, Yates C, You Z, Tan M (2018). Diverse roles of mitochondria in immune responses: novel insights into Immuno-metabolism. Front Immunol.

[25] Cai S, Ou Z, Liu D, Liu L, Liu Y, Wu X, Yu T, Peng J (2018). Risk factors associated with liver steatosis and fibrosis in chronic hepatitis B patient with component of metabolic syndrome. United European Gastroenterol J.

[26] Cai S, Yu T, Jiang Y, Zhang Y, Lv F, Peng J (2016). Comparison of entecavir monotherapy and de novo lamivudine and adefovir combination therapy in HBeAg-positive chronic hepatitis B with high viral load: 48-week result. Clin Exp Med.

[27] Cheng CT, Kuo CY, Ouyang C, Li CF, Chung Y, Chan DC, Kung HJ, Ann DK (2016). Metabolic stress-induced phosphorylation of KAP1 Ser473 blocks mitochondrial fusion in breast Cancer cells. Cancer Res.

[28] Kannan A, Wells RB, Sivakumar S, Komatsu S, Singh KP, Samten B, Philley JV, Sauter ER, Ikebe M, Idell S (2016). Mitochondrial reprogramming regulates breast Cancer progression. Clin Cancer Res.

[29] Yan H, Qiu C, Sun W, Gu M, Xiao F, Zou J, Zhang L (2018). Yap regulates gastric cancer survival and migration via SIRT1/Mfn2/mitophagy. Oncol Rep.

[30] Zhang GE, Jin HL, Lin XK, Chen C, Liu XS, Zhang Q, Yu JR (2013). Anti-tumor effects of Mfn2 in gastric cancer. Int J Mol Sci.

